# Supplementary figures and images for: Effects of diet and gizzard muscularity on grit use in domestic chickens
Source: PeerJ. 2020 Nov 12;8:e10277. doi: 10.7717/peerj.10277 (PMC7666813; doi:10.7717/peerj.10277)

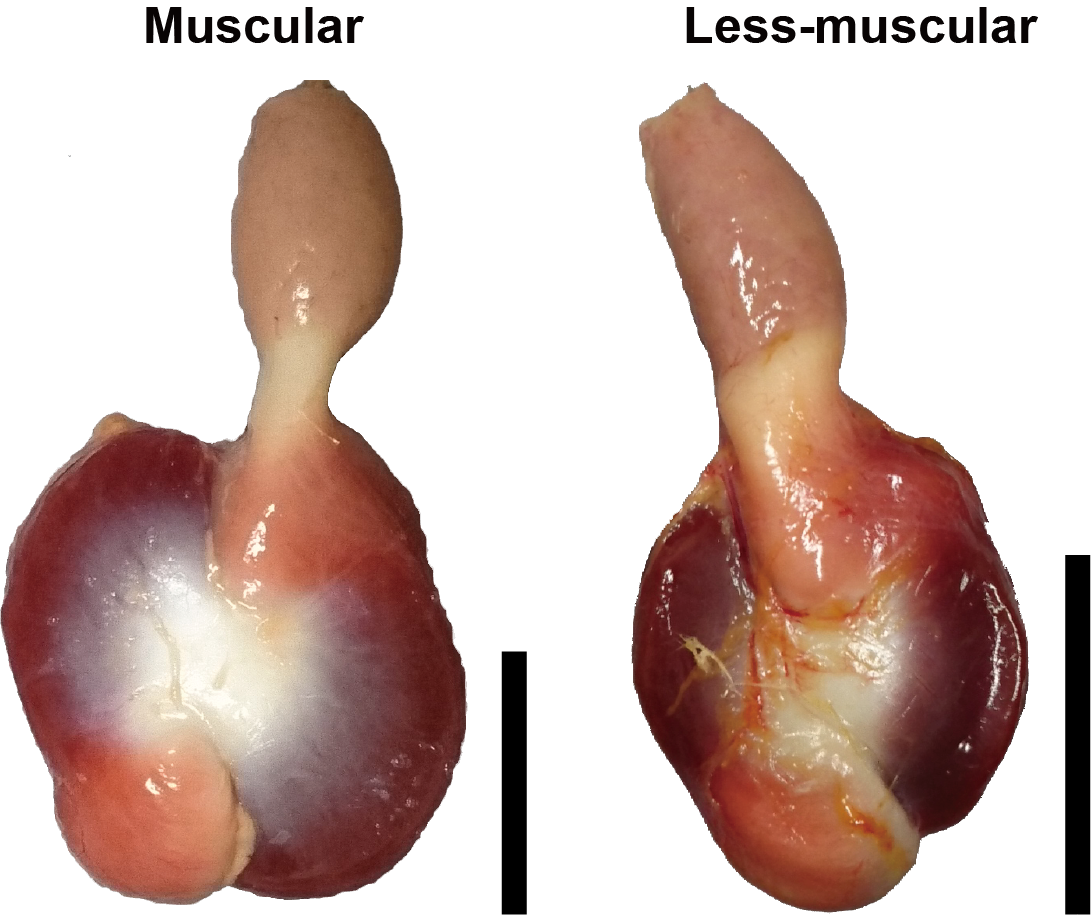

Supplement: Supplemental Information 7 — Scales = 2 cm. [file peerj-08-10277-s007.png]

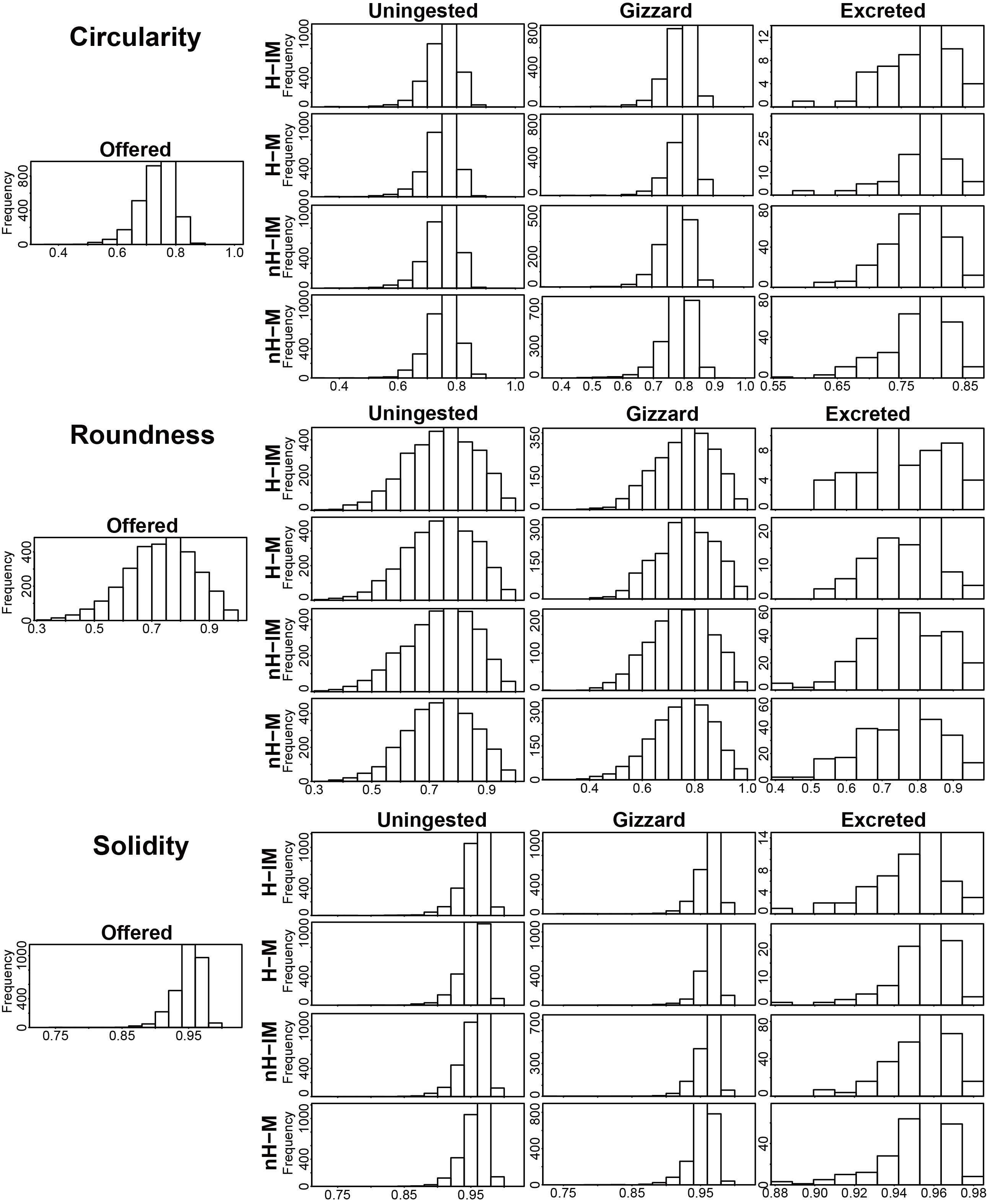

Supplement: Supplemental Information 8 — Abbreviations: H-lM, herbivorous diet with a less-muscular gizzard; H-M, herbivorous diet with a muscular gizzard; nH-lM, non-herbivorous diet with a less-muscular gizzard; nH-M, non-herbivorous diet with a muscular gizzard. [file peerj-08-10277-s008.png]
